# Optimization of Low-Cost Ti-35421 Titanium Alloy: Phase Transformation, Bimodal Microstructure, and Combinatorial Mechanical Properties

**DOI:** 10.3390/ma12172791

**Published:** 2019-08-30

**Authors:** Fuwen Chen, Guanglong Xu, Yuwen Cui, Hui Chang

**Affiliations:** 1 Tech Institute for Advanced Materials & College of Materials Science and Engineering, Nanjing Tech University, Nanjing 210009, China; 2 School of Materials Science and Engineering, Central South University, Changsha 410083, China

**Keywords:** Ti–35421, β ↔ α transformation, thermo-kinetics, bimodal microstructure, fracture toughness

## Abstract

A sophisticated understanding of phase transformations and microstructure evolution is crucial in mechanical property optimization for the newly developed low-cost Ti-35421 (Ti-3Al-5Mo-4Cr-2Zr-1Fe wt.%) titanium alloy. The phase transformations in dual-phase Ti-35421 were studied by experiments and thermo-kinetic modeling. The phase transformation reactions and temperature ranges were determined as β→α_lamellar_ [410–660 °C], α_lamellar_→β [660–740 °C], α_lath_→β [740–825 °C]. The Gibbs-Thomson effect and multicomponent diffusivities were proven to be responsible for the distinguishing behaviors of growth and dissolution between two α phases. The aging temperature of 540 °C was optimized based on calculations. It introduced a bimodal microstructure containing stubby α lamellae and β matrix. The mechanical properties of bimodal Ti-35421 were tested and compared with baseline alloy Ti-B19 and other near-β titanium alloys. The 540 °C aged alloy exhibits an optimal combination of mechanical properties with tensile strength of 1313 MPa, yield strength of 1240 MPa, elongation of 8.62%, and fracture toughness of 75.8 MPa·m^1/2^. The bimodal Ti-35421 shows comparable performance to Ti-B19 but has lower cost in raw materials and processing. The results also demonstrate that thermo-kinetic modeling can effectively be utilized in tailoring microstructure and enhancing mechanical properties.

## 1. Introduction

Near-β titanium alloys have already been applied in the aerospace industry to fabricate landing gears and engine parts [1,2,3,4,5]. However, the main impediment to the widespread use of titanium alloys is the soaring cost in raw materials and processing. The cost comprises the costly extraction of pure Ti, the multi-pass melting and thermo-mechanical processing, the high waste rate, and the expensive alloying elements like V, Nb, Mo, etc. [6,7,8,9]. Therefore, great efforts have been made to reduce the cost of titanium alloys, meanwhile to maintain or improve strength, ductility, and fracture toughness. Fe is a common element which is much cheaper than V, Nb, and Mo and has the ability to stabilize β phase. A few Fe alloyed near-β titanium alloys, such as Ti-1.5 Al-6.8 Mo-4.5 Fe, have been developed and demonstrated excellent mechanical properties [10,11,12]. Moreover, the addition of Fe facilitates thermo-mechanical processing by reducing flow stress [13], thus saves heat and electric consumption. In brief, the alloying of Fe can reduce the costs of raw materials and processing in titanium industry. 

Ti-3Al-5Mo-4Cr-2Zr-1Fe (Ti-35421, wt.%) alloy was a newly developed low-cost near-β titanium alloy with Ti-3Al-5Mo-5V-4Cr-2Zr (Ti-B19, wt.%) [14] as the baseline alloy. The chemical composition of Ti-35421 was designed by replacing 5% V in Ti-B19 with 1% Fe and keeping the Mo equivalent unchanged. Due to the β-stabilizing effect, the synergy among Mo, Cr, and Fe provided solid-solution strengthening and ductility of β phase. The chemical composition of Al was unchanged to maintain the strengthening from the precipitation of α phase. Zr is neutral element which can improve hot working property of the alloy. Thus, the product prototype of Ti-35421 already has high strength of 900 to 1450 MPa, and moderate ductility of 6–15% [15]. Similar to other near-β titanium alloys, the mechanical properties of Ti-35421 fluctuated with amount and morphology of α phase, showing a large adjustable window. The industrial application of Ti-35421 alloy requires good combinatorial mechanical properties. It refers to a combination of high/acceptable strength, ductility, and fracture toughness. Solid-solution Treatment and Aging (STA) is a commonly used heat treatment in near-β titanium alloys. It consists of solid-solution treatment in the α + β phase region followed by cooling to the target temperature in the α + β phase region and then aging at the final temperature. It is capable of introducing the bimodal microstructure in which the fine lamellar α phase enhances strength [16,17]; the coarse plate or equiaxed α is beneficial to ductility [18]; and the synergy of two α phases and β matrix might boost fracture toughness [19,20]. However, the quantitative description of microstructure development and mechanical performance is still pending. A deep understanding of the phase transformations and microstructural modifications occurring during STA are thus required to allow the improvement of the mechanical performances of Ti-35421.

In this work, aging temperature in STA and combinatorial mechanical properties are optimized based on the thermo-kinetic investigation of growth and dissolution of α phases. For this purpose, the phase transformation reactions and temperature ranges in the alloy after solid-solution treatment are studied by combing dilatometry, microstructure observation, and thermo-kinetic modeling. The growth and/or dissolution behaviors of α_lamellar_ and α_lath_ are quantitatively described. The temperature dependent growth rate is also predicted. Whereafter, it is to observe the microstructure and test the mechanical properties of alloy aged at the selected temperature. The effect of STA aging temperature on microstructure development and mechanical properties is discussed. 

## 2. Materials and Methods 

### 2.1. Material

The Ti-35421 alloy ingot was fabricated by vacuum consumable arc melting three times. The raw materials were grade 0_A_ sponge Ti (Ti wt.% > 99.8%), high purity Al (99.9 wt.%), Cr (99.9 wt.%), Fe (99.9 wt.%) and Zr (99.9 wt.%), and Al-80Mo master alloys. The chemical composition of the ingot was determined by titration. The actual chemical composition is listed in Table 1, showing small deviation from the nominal chemical composition. A slab with 74 mm in thickness was formed from the as-cast ingot via multi-pass forging in the β phase region. The forged slab was then hot rolled in the α + β phase region down to 22 mm in thickness. It was confirmed by the metallographic method that the β-transus temperature was approximately 815 ± 10 °C. The obtained rolled sheet was subjected to solid-solution treatment at 780 °C for 1 h followed by air cooling to obtain a dual-phase microstructure of β + α_lath_. This preset microstructure was employed as initial microstructure in this work. The chemical compositions of β and α_lath_ phases were detected by an Electron Probe Micro-Analyzer (EPMA, JEOL JXA 8900, Tokyo, Japan). 

### 2.2. Dilatometric Test

The temperatures of phase transformation reactions which take place in the β + α_lath_ alloy were determined by dilatometric test on Netzsch^®^ DIL-402C (Selb, Germany). The tests were carried out under an argon atmosphere (purity ≥ 99.99%) from room temperature to 1000 °C at a heating rate of 1 °C/min. The specimens were wire cut from the solid solution-treated alloy and polished into Ф 5 mm × 25 mm cylinders. The phase transformation temperature and length change of specimens were recorded by sensors to plot the dilatometric curve.

### 2.3. Heat Treatment

The specimens with initial microstructure were heat treated following two schemes for different purposes. The first scheme was continuously heating to the temperatures of the dilatometric readings with the same rate of 1 °C/min followed by a water quench. The heat-treated specimens were reserved for microstructure observation and to figure out the reactions of phase transformation corresponding to the dilatometric signals. The second scheme was 8 h aging at temperatures in different metastable phase regions followed by air cooling. It was to optimize the mechanical properties as the responses to different aging temperatures. Both heating and isothermal heat treatments were carried out in a Carbolite GVA 12/450 vacuum furnace (Hope, UK).

### 2.4. Microstructure Observation

The heat-treated microstructure of Ti-35421 was observed by Scanning Electron Microscopy (SEM) on NOVA^TM^ Nano SEM 230 (FEI, Hillsboro, OR, USA). The specimens for SEM observation were mechanically polished with SiC paper and diamond paste (1 μm), and then with colloidal silica (OP-S). The finished specimens showed a mirror-like surface. With the SEM images, the phase size and fraction were obtained by using Image-Pro Plus software (Media Cybernetics, Inc., Rockville, MD, USA). The phase size and fraction were repetitively measured in different areas, thus the average values of phase fraction, length, and width of lath and lamellar α phases were of statistical significance. The phase constitution were detected on the Bruker D8 (Billerica, MA, USA) X-Ray Diffractometer using Cu Kα radiation (λ = 1.5406 Å). It was operated at 40 kV, 40 mA with the scanning rate of 4°/min and 2θ range of 30°–65°. 

### 2.5. Mechanical Properties

Ultimate tensile strength, yield tensile strength, and elongation of the alloy were examined on an ETM205D (Wance, Shenzhen, China) testing machine with a nominal strain rate of 1 mm/min at room temperature. Standard cylindrical tensile specimens were cut out parallel to the rolling direction (RD) by wire electric discharge machining, a gauge length and diameter being 100 mm and 10 mm, according to ASTM E8/E8M–09 [21]. The measurement of Mode-I fracture toughness in the condition of plane strain, *K_IC_*, was performed on an MTS 370 (Eden Prairie, MN, USA) with an electro-hydraulic servo system. The chevron-notched half compact-tension specimens with the thickness of 20 mm were wire cut with L-T (rolling-long transverse) orientations from the plates and machined as per ASTM standard E399-06 [22]. The opening direction of the pre-crack was selected paralleling to the long transverse direction, and the direction of crack propagation was paralleling to the rolling direction. The fracture toughness was calculated using the formula given in the ASTM standard E399, and the final value of mode-I fracture toughness was obtained by averaging the testing values of three measurements.

## 3. Results and Discussions

### 3.1. Initial Microstructure of Solid Solution Treated Alloy

**Figure 1:**
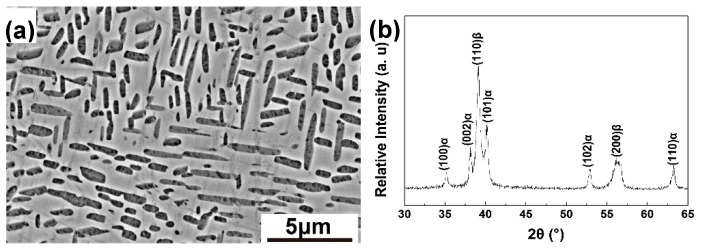
(**a**) Back-scatter Scanning Electron Microscopy (SEM) image and (**b**) XRD pattern of Ti-35421 after solid solution.

The dual-phase microstructure and XRD pattern of Ti-35421 after solid-solution treatment is shown in Figure 1. The dark grey α_lath_ phase accounting for 30.8% in volume fraction is embedded in the light grey β matrix. The α_lath_ phase shows anisotropic morphology, with an average length of 1.37 μm and an average width of 0.42 μm. The chemical composition of β phase is Ti-4.69 Al-2.80 Mo-3.00 Cr-0.87 Zr-0.62 Fe (at. %), and that of α_lath_ phase is Ti-5.99 Al-1.62 Mo-1.79 Cr-0.81 Zr-0.25 Fe (at. %).

### 3.2. Phase Transformation Reactions and Temperature Ranges

**Figure 2:**
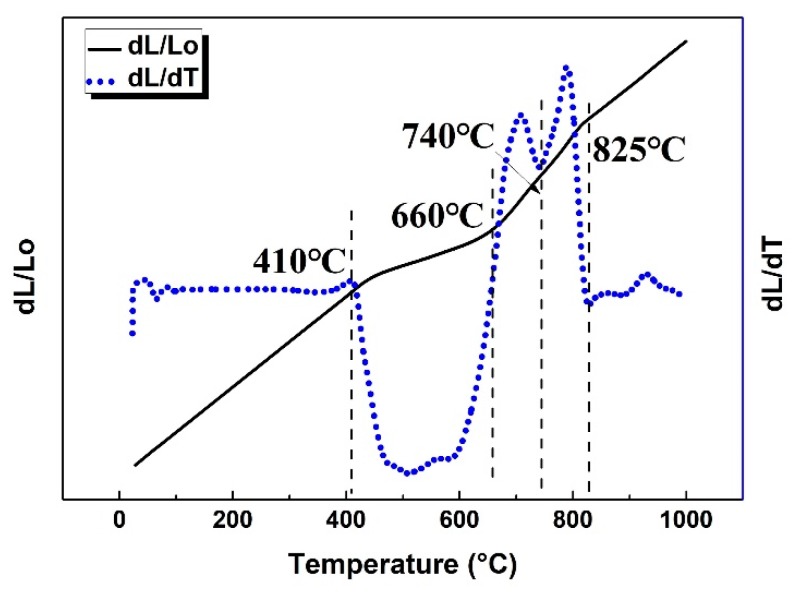
Dilatometric curve (in solid) and the derivative curve (dotted curve) at 1 °C/min during the continuous heating process.

The phase transformation reactions and their temperature ranges were determined by combining dilatometry and microstructure observation. Figure 2 shows the dilatometric curve and its derivative curve at the heating rate of 1 °C/min. There are three peaks or valleys deviating from the horizontal line of the derivative curve, which indicate the occurrence of phase transformations: (1) negative deviation from 410 to 660 °C; (2) positive deviation from 660 to 740 °C; (3) positive deviation from 740 to 825 °C. 

**Figure 3:**
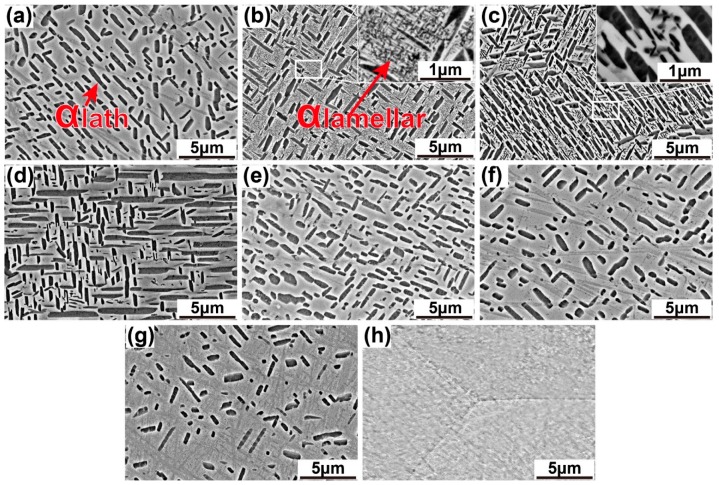
SEM images of the microstructure at (**a**) 410, (**b**) 550, (**c**) 660, (**d**) 700, (**e**) 740, (**f**) 780, (**g**) 800, and (**h**) 840 °C during 1 °C/min heating.

**Figure 4:**
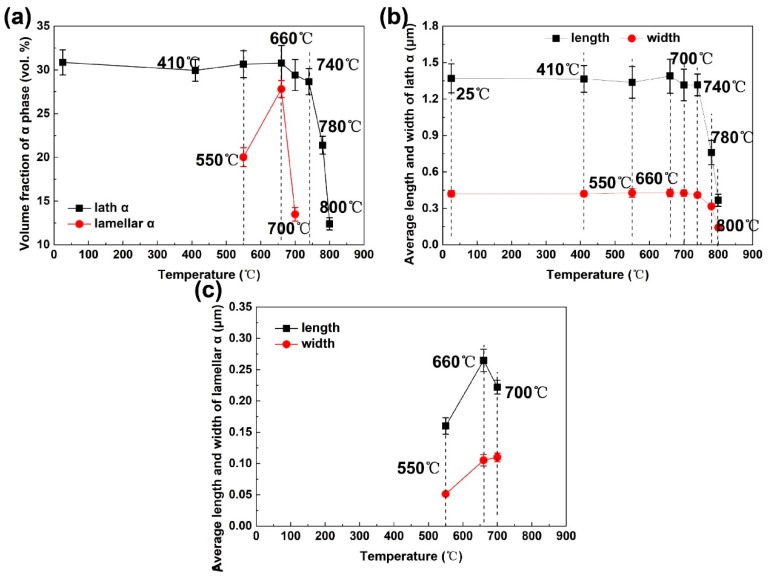
The variations of (**a**) volume fraction of lath and lamellar α phases, (**b**) average length and width of lath α, and (**c**) average length and width of lamellar α with temperatures.

To identify the phase transformation reactions occurring at the temperatures of dilatometric readings, the specimens were heated to 410, 550, 660, 700, 740, 780, 800, and 840 °C at 1 °C/min individually followed by quench for microstructure observation and phase identification (see Figure 3). In the SEM images, the α_lath_ phase is almost unchanged in size and volume fraction between 410 and 740 °C (Figure 3a–d), yet undergoes dissolution from 740 to 840 °C (Figure 3e–h). There is α_lamellar_ phase showing differences in size and volume fraction from α_lath_. It experienced nucleation, growth, and dissolution during the heating, indicating by the changes of lengths and widths of α_lamellar_. The α_lamellar_ phase starts to precipitate in β matrix at 410 °C, followed by a growth in length and width between 550 and 660 °C (Figure 3b,c). Up to 660 °C, the α_lamellar_ phase begins to dissolve back to β phase, exhibiting decreases in volume fraction and length as well as a slight increase in average width (Figure 3d). At 740 °C, the α_lamellar_ phase disappears in the SEM image, indicating completion of the dissolution (Figure 3e). Above 740 °C (Figure 3f,g), the α_lath_ shows obvious decrease in length, width, and volume fraction. It is completely dissolved back into β matrix at 840 °C (Figure 3h). The alloy thus shows a single β phase microstructure at 840 °C. The variations of average lengths, average widths, and phase fractions of α_lath_ and α_lamellar_ phases are summarized in Figure 4 and Table 2. Therefore, the phase transformation reactions of dual-phase Ti-35421 and their temperature ranges are determined β→α_lamellar_ [410–660 °C], α_lamellar_ →β [660–740 °C], α_lath_→β [740–825 °C].

### 3.3. Thermo-Kinetic Modeling of Growth and Dissolution

With the discrete experimental data in Section 3.2, thermo-kinetic modeling was implemented to evaluate growth and dissolution of α phases in continuous functions, and then to determine the optimal temperature which can introduce the bimodal microstructure of α_lath_ and dispersed α_lamellar_. Following the strategy of previous work of present authors [23], different models were applied to lengthening of α_lamellar_ tip, dissolution of α_lamellar_ tip, thickening of α_lamellar_ (broad face of α_lamellar_), and dissolution of α_lath_ (broad face of α_lath_) based on the following assumptions. (1) The local equilibrium condition at each time/temperature step was approximately satisfied at the interface; (2) the Gibbs-Thomson effect played a part in lengthening/dissolution of the α_lamellar_ tip because of the tip curvature, but did not work in thickening/dissolution of the broad face. The details of the modeling can be found in Appendix A and in [24,25,26]. By selecting the appropriate diffusion coefficients from the handbook [27], the interface migration distances in calculations were compared with the experiments. Then, the interface migration rate was calculated. The specific diffusion mechanism which governed growth/dissolution of the α phases were determined. 

**Figure 5:**
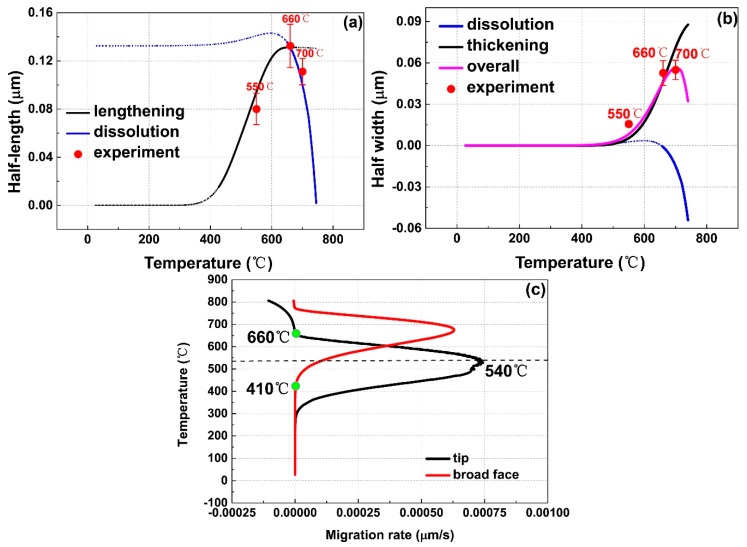
(**a**) Calculated growth (black) and dissolution (blue) curves for α_lamellar_ tip; (**b**) thickening (black), dissolution (blue), and real interface migration (magenta) curves for α_lamellar_ broad face are compared with the measured average length and width of α lamellae at different temperatures (red disc). (**c**) The calculated migration rates of tip and broad face in lamellar α phase.

Figure 5a shows the migration distances of α_lamellar_ tip by using lengthening and dissolution models, individually. The sigmoid shaped curve in black predicts the lengthening of α_lamellar_ tip, which integrated the lengthening rate based on the modified Ivantsov equation with the Gibbs-Thomson effect. The curve in blue indicates the dissolution of α_lamellar_ tip, which was obtained by implementing an integral of Aaron’s dissolution rate equation together with the Gibbs-Thomson calibration. The two curves intersect at 661 °C, coincided with the onset dissolution temperature of α_lamellar_ in the experiment. Thus, the curve in solid describes the real migration distances of α_lamellar_ tip. The calculated lengthening distances are in good agreement with half values of the α_lamellar_ length at 550, 660, and 700 °C in the experiment, considering that an α lamella has two tips. More important is that the lengthening/dissolution rate of α_lamellar_ tip has been calculated and shown in Figure 5c, which is unable to be directly extracted from the experiment. The shape of the curve is similar to that in Porter’s textbook [25]. The low lengthening rate is attributed to the small diffusion coefficients at low temperature and the small supersaturation at high temperature. The calculated rate shows a maximum at 540 °C. It is located in the deepest valley on the derivative dilatometric curve (Figure 2). The temperature with high lengthening rate and observable thickening rate will be employed as the base to optimize the bimodal microstructure and mechanical properties in Section 3.4. Above 661 °C, the rates show negative values, indicating the occurrence of dissolution.

Figure 5b depicts thickening and shrinkage of the broad face of α_lamellar_. The thickening curve of the broad face (in black) shows a similar sigmoid shape as that of α_lamellar_ tip. The difference lies in that the onset of thickening is shifted to higher temperature due to the absence of the Gibbs-Thomson effect. The interface migration distances at 550 and 660 °C predicted by the single coarsening model are good enough to be compared with half widths of α_lamellar_. However, it is not true for that at higher temperatures.At 700 °C, the dissolution of α_lamellar_ tip due to the Gibbs-Thomson effect would give rise to an additional diffusion flux along the broad face. Thus, both dissolution of α_lamellar_ tip and thickening of α_lamellar_ broad face do substantial contributions to the formation of stubby α lamellae. The summation of both effects determines a parabolic-like kinetic curve illustrated in magenta above 660 °C. Additionally, the half-width of α_lamellar_ phase predicted by the two-factor kinetics coincides with the experimental value at 700 °C. In Figure 5c, the lengthening/dissolution rate of α_lamellar_ broad face also exhibits the bell shape. The difference of onset temperatures between broad face thickening and tip lengthening is more clearly shown, which is crucial in tailoring the aspect ratio of α lamella.

**Figure 6:**
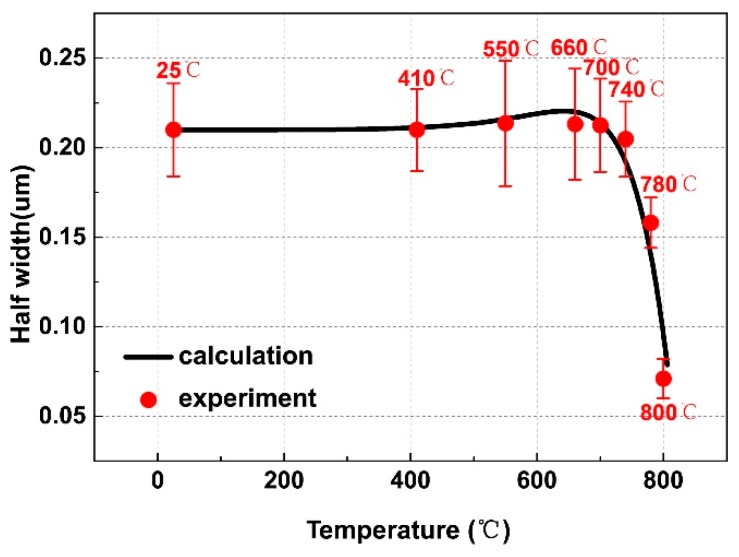
Calculated kinetic curves describe negligible growth and remarkable re-dissolution of α lath. The calculation results are compared with average half-width of lath α phase in the experiment.

The dissolution of α_lath_ broad face follows simple kinetics of planar interface due to the large curvature at the α_lath_ tip and the negligible Gibbs-Thomson effect. Figure 6 illustrates the dissolution curve of α_lath_ broad face. The measured average widths in the entire temperature range are well represented by the calculation. 

### 3.4. Mechanical Properties Dependent on Aging Temperature and Microstructure Features

Since the α_lath_ phase hardly changed in the temperature range of 410–740 °C, mechanical properties optimization of the bimodal Ti-35421 alloy would mainly depend on the microstructure tailoring for the α_lamellar_ phase. The aging temperature then plays important roles in determining α_lamellar_ size and volume fraction. In Figure 5c, the calculated lengthening rate of α_lamellar_ tip has the maximum value at 540 °C, and the thickening rate of α_lamellar_ broad phase has a moderate value. It infers that aging at temperatures around 540 °C is capable of introducing more dispersed α_lamellar_ in a shorter time. Meanwhile, α_lamellar_ tip and broad face can grow in an appropriate velocity ratio. Therefore, the isothermal aging has been carried out at 500, 520, 540, and 560 °C, respectively, followed by the tests of mechanical properties. The aging time was set to 8 h which ensured the completion of α_lamellar_ precipitation at all temperatures and avoided ripening as far as possible, so that the relationship between mechanical properties, microstructure features, and aging temperatures could be studied. 

**Figure 7:**
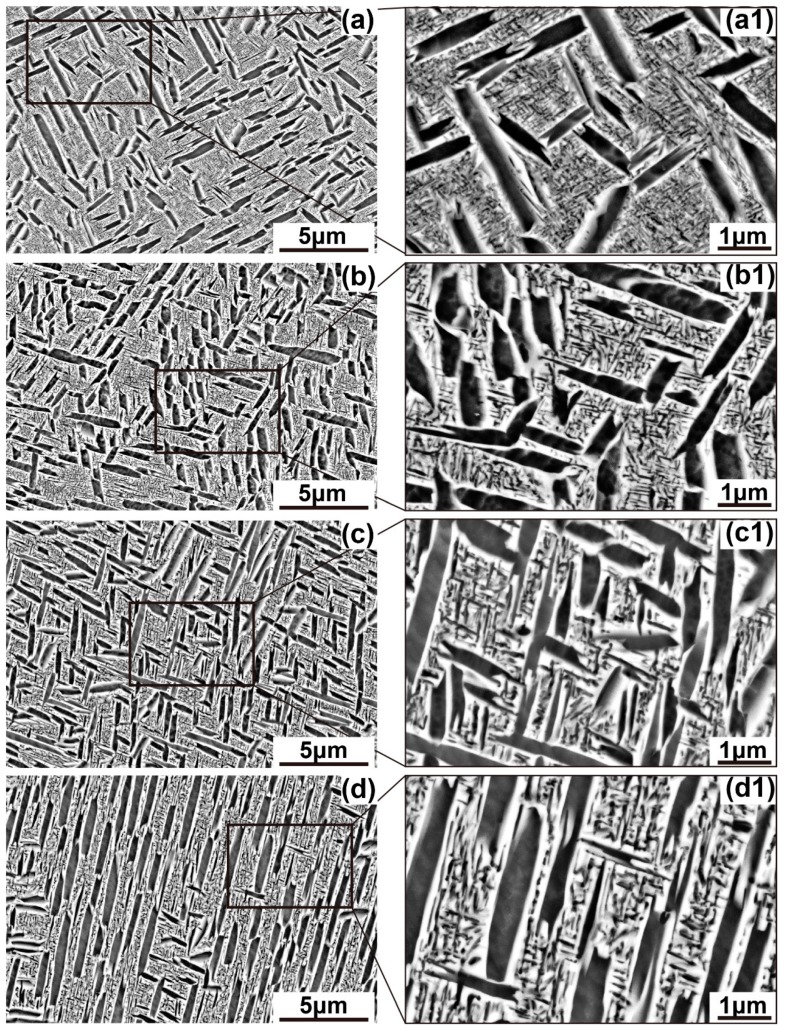
SEM images of bimodal microstructure after aging for 8 h: (**a**,**a1**) 500 °C, (**b**,**b1**) 520 °C, (**c**,**c1**) 540 °C, and (**d**,**d1**) 560 °C.

**Figure 8:**
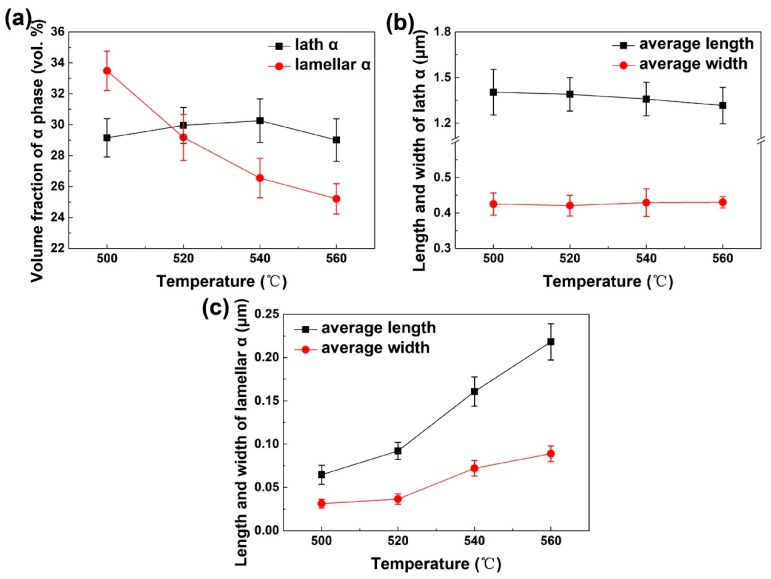
The variations of (**a**) volume fraction of lath and lamellar α phases, (**b**) average length and width of lath α, and (**c**) average length and width of lamellar α with temperatures.

The bimodal microstructure of Ti-35421 after aging is displayed in Figure 7. The microstructure features of α_lamellar_ and α_lath_ phases were extracted from SEM images. The variations of microstructure features with aging temperatures are plotted in Figure 8; and the data are summarized in Table 3. There are only slight changes in average length, width, and volume fraction of α_lath_ phase with the variation of aging temperatures. It is contrasted with the remarkable changes in α_lamellar_. The volume fraction of α_lamellar_ precipitates decreases as the aging temperature increases. However, the average length of α lamellae is monotonically increasing from 500 °C to 560 °C. The average width of α_lamellar_ at 500 °C is almost identical to that at 520 °C, much smaller than that at 540 °C and 560 °C. 

**Figure 9:**
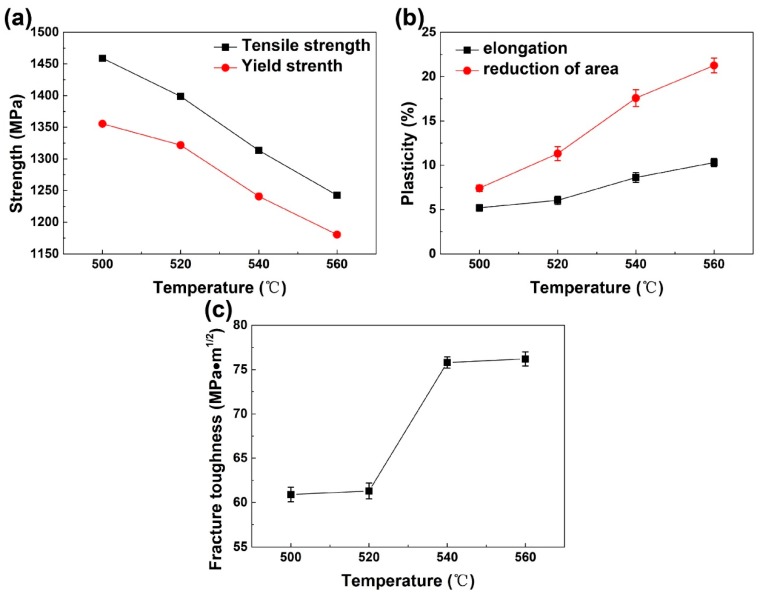
The variations of mechanical properties with aging temperatures: (**a**) strength, (**b**) plasticity, and (**c**) fracture toughness.

The results of mechanical property tests demonstrate the bimodal Ti-35421 has a wide performance window by tailoring the microstructure and adjusting the aging temperatures. The ultimate tensile strength, yield tensile strength, elongation, reduction of area, and Mode I fracture toughness (*K_IC_*) can be achieved in a range of 1240.52–1459.03 MPa, 1180.40–1355.54 MPa, 5.20–10.31%, 7.42–21.26%, and 60.9–76.2 MPa·m^1/2^, respectively. Figure 9 depicts the mechanical property variations of aged Ti-35421 with the aging temperatures. And the data are summarized in Table 4. The trade-off between strength and plasticity is clearly shown by comparing Figure 9a,b. The tensile strength and yield strength of the bimodal alloy decrease dramatically with the increase of aging temperature. The elongation and area reductions are on the contrary, which monotonously increase along with the increased aging temperature. *K_IC_* shows different temperature dependence from other properties. It exhibits a jump from 520 to 540 °C and two platform values at 500–520 °C and 540–560 °C. 

**Figure 10:**
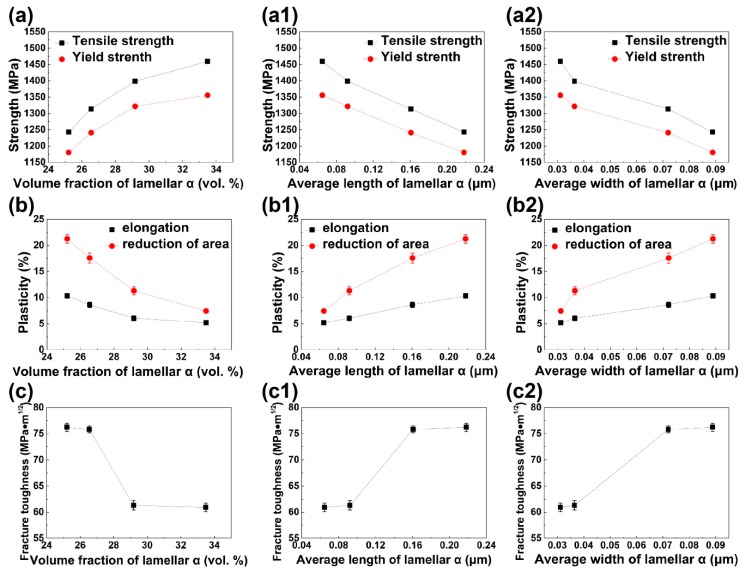
The variations of (**a**, **a1**, **a2**) strength, (**b**, **b1**, **b2**) plasticity, and (**c**, **c1**, **c2**) fracture toughness with microstructure features of (**a**–**c**) volume fraction of α_lamellar_, (**a1**–**c1**) average length of α_lamellar_, and(**a2**–**c2**) average width of α_lamellar_.

Figure 10 depicts the dependency relationships between microstructure features and mechanical properties. The more α_lamellar_ in smaller size is beneficial to strength but reduces the performance of plasticity. The microstructure dependent *K_IC_* also exhibits an approximate step function shape on the bottom row of Figure 10. It infers that the fracture toughness of bimodal Ti-35421 is closely linked to the kinetics of α_lamellar_ growth/dissolution at different temperatures. As is demonstrated in Figure 5c, the migration rate of α_lamellar_ broad face is low in comparison with that of α_lamellar_ tip at 500–520 °C. The elongated fine α_lamellar_ might introduce the stress concentration at the lamella tips. Due to the dispersed distribution of the α_lamellar_, the overlap of stress concentration zones would be detrimental to the fracture toughness. Above 540 °C, the migration rate of α_lamellar_ broad face dramatically increases, therefore, the α_lamellar_ tip and broad face can grow in an appropriate ratio. The reduction in the aspect ratio of α_lamellar_ results in optimum balance of strength, ductility, and fracture toughness [28]. Therefore, the optimal combinatorial mechanical performance was obtained in the alloy aged at 540 °C, especially considering the damage tolerance.The ultimate tensile strength, yield strength, elongation, reduction of area, and fracture toughness were measured 1313.39 MPa, 1240.81 MPa, 8.62%, 17.58%, and 75.8 MPa·m^1/2^, respectively.

**Figure 11:**
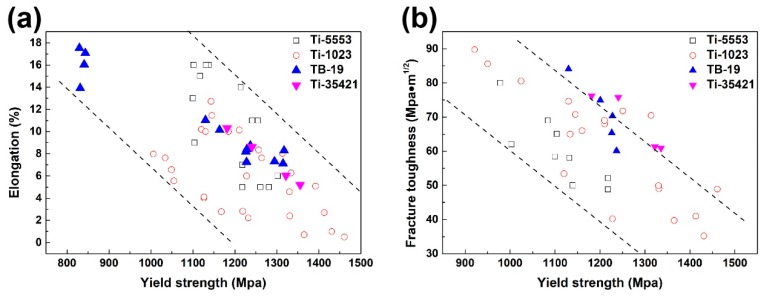
Performance comparison between Ti-35421, Ti-B19, Ti-5553, and Ti-1023: (**a**) yield strength vs. elongation, (**b**) yield strength vs. fracture toughness. (Ti-B19 [29], Ti-5553 [30,31], Ti-1023 [19,32]).

Figure 11 compares the combinatorial mechanical performance of Ti-35421 at the above aging temperatures with Ti-B19 [29] and other conventional near-β Ti alloys [19,30,31,32]. Here, the combinations of elongation/yield strength and elongation/fracture toughness were selected as the representatives. Under the same performance of the yield strength, Ti-35421 is comparable to Ti-B19 in ductility and Mode I fracture toughness. It demonstrated that the substitution of Fe for V not only lowered the cost of raw materials but maintained the mechanical performances. Thus, the new developed Ti-35421 is promising in marine and aeronautic applications.

## 4. Conclusions

A low-cost Ti-3Al-5Mo-4Cr-2Zr-1Fe (Ti-35421) alloy was prepared with the baseline alloy of TB-19. The expensive alloying element V was substituted by the lower-priced element Fe. The phase transformations in Ti-35421 alloy with the preset microstructure of β + α_lath_ were studied by combing experiments and thermo-kinetic modeling. The phase transformation reactions and temperature ranges were determined as (1) β→α_lamellar_ [410–660 °C], (2) α_lamellar_→β [660–740 °C], (3) α_lath_→β [740–825 °C]. The Gibbs-Thomson effect and multicomponent diffusion coefficients played important roles in the growth/dissolution of α_lamellar_ phase. The optimized aging temperature of 540 °C was obtained based on the calculation. It was capable of introducing bimodal microstructure with stubby α_lamellar_ phase. The mechanical properties of bimodal Ti-35421 were tested and compared with Ti-B19 and other near-β titanium alloys. The 540 °C aged bimodal alloy exhibited optimal combinatorial mechanical properties of ultimate tensile strength of 1313.39 MPa, yield strength of 1240.81 MPa, elongation of 8.62%, and Mode I fracture toughness of 75.8 MPa·m^1/2^. The performance is comparable to two commercial near-β titanium alloys. The results demonstrated that it is feasible to reduce the cost of titanium alloys by substituting Fe for V. It was also manifested that thermo-kinetic modeling can effectively be utilized in tailoring microstructure and enhancing mechanical properties.

## Figures and Tables

**Table 1:** Actual chemical composition of the Ti-35421 alloy by titration.

Ti/wt.%	Al/wt.%	Mo/wt.%	Cr/wt.%	Zr/wt.%	Fe/wt.%	O/wt.%
Balance	3.03	5.02	3.80	1.87	0.995	0.087

**Table 2:** Microstructure features of α phases quenched from different temperatures during 1 °C/min heating.

Temperature/°C	α_lath_			α_lamellar_		
	Average Length/μm	Average Width/μm	Volume Fraction/vol. %	Average Length/μm	Average Width/μm	Volume Fraction/vol. %
25	1.370 ± 0.120	0.420 ± 0.026	30.86 ± 1.43			
410	1.373 ± 0.112	0.420 ± 0.023	29.95 ± 1.25			
550	1.341 ± 0.133	0.427 ± 0.035	30.66 ± 1.54	0.160 ± 0.013	0.031 ± 0.002	20.02 ± 1.09
660	1.389 ± 0.144	0.426 ± 0.031	30.78 ± 2.01	0.265 ± 0.018	0.105 ± 0.009	27.81 ± 0.98
700	1.320 ± 0.143	0.425 ± 0.026	29.41 ± 1.78	0.222 ± 0.011	0.109 ± 0.007	13.49 ± 0.78
740	1.321 ± 0.096	0.409 ± 0.021	28.65 ± 1.49			
780	0.764 ± 0.101	0.316 ± 0.021	21.39 ± 1.02			
800	0.372 ± 0.053	0.142 ± 0.011	12.40 ± 0.69			

**Table 3:** Microstructure features of α phases after aging for 8 h.

Temperature/°C	α_lath_			α_lamellar_		
	Average Length/μm	Average Width/μm	Volume Fraction/vol. %	Average Length/μm	Average Width/μm	Volume Fraction/vol. %
500	1.404 ± 0.151	0.425 ± 0.051	29.16 ± 1.24	0.065 ± 0.011	0.031 ± 0.005	33.49 ± 1.27
520	1.390 ± 0.112	0.421 ± 0.049	29.96 ± 1.17	0.092 ± 0.010	0.037 ± 0.006	29.18 ± 1.49
540	1.359 ± 0.11	0.429 ± 0.056	30.26 ± 1.41	0.161 ± 0.017	0.072 ± 0.009	26.55 ± 1.28
560	1.316 ± 0.12	0.430 ± 0.061	29.01 ± 1.38	0.218 ± 0.021	0.089 ± 0.009	25.21 ± 0.98

**Table 4:** Mechanical properties of the alloy after aging for 8 h.

Temperature/°C	Strength		Plasticity		Fracture Toughness *K_IC_*/MPa·m^1/2^
	Tensile Strength *σ_s_*/MPa	Yield Strength *σ_y_*/MPa	Elongation *δ*/%	Reduction of Area *ψ*/%	
500	1459.03 ± 4.36	1355.54 ± 4.67	5.20 ± 0.38	7.42 ± 0.37	60.9 ± 0.82
520	1398.66 ± 3.23	1321.74 ± 4.82	6.04 ± 0.47	11.31 ± 0.78	61.3 ± 0.89
540	1313.39 ± 3.48	1240.81 ± 4.98	8.62 ± 0.56	17.58 ± 0.96	75.8 ± 0.64
560	1242.52 ± 3.94	1180.40 ± 3.67	10.31 ± 0.46	21.26 ± 0.83	76.2 ± 0.79

## Appendix A

### A.1. Lengthening Model of α_lamellar_ Tip

The modified Ivantsov equation, Equation (A1), with the Gibbs-Thomson calibration was employed to calculate the average lengthening rate of α_lamellar_ tip.

(A1) $$\Omega =\sqrt{\pi P}\exp\left(1-\mathrm{erf}\sqrt{P}\right)\left(1+\frac{\rho_{c}}{\rho }\Omega \cdot S\left(P\right)\right)$$

The demanded lengthening rate *v* is embedded in the Peclet number *P*.

(A2) $$P=\frac{v\rho }{2D}$$

The Peclet number indicates the ratio of convection and diffusion. The tip radius *ρ* is time and temperature dependent. For simplicity, *ρ* was calculated under the equilibrium condition:

(A3) $$\rho =\frac{2\sigma V_{m}}{\mu_{Ti}^{\beta }-\mu_{Ti}^{\alpha }}.$$

Interfacial energy *σ* was assumed 0.2 J/m^2^. *V_m_* is molar volume of the alloy which was estimated following Vegard’s law. $\mu_{Ti}^{\alpha }$ and $\mu_{Ti}^{\beta }$ are the chemical potentials of Ti in α and β phases, respectively. *D* is the diffusion coefficient. For a multicomponent and multiphase system, an appropriate *D* should be selected in different kinetic cases. In the lengthening of α_lamellar_ tip, the diffusion coefficient of α phase was employed. It was averaged over the chemical diffusion coefficients of partitioned elements.

In the last parenthesis of Equation (A1), the second term was introduced by Trivedi [33,34] to calibrate the effect of interfacial curvature on the local composition. *ρ_c_* is the critical radius for growth and was set to 9.0 × 10^−10^ m. *S*(*P*) was approximated as *2/πP* in accord with [24]. *Ω* is the non-dimensional supersaturation of each partitioned element and expressed as

(A4) $$\Omega =\frac{x_{0}^{\beta }-x^{\beta /\alpha }}{\sqrt{\left(x^{\alpha /\beta }-x_{0}^{\beta }\right)\left(x^{\alpha /\beta }-x^{\beta /\alpha }\right)}}.$$

$x_{0}^{\beta }$ is the mole fraction of alloying element in β phase before α_lamellar_ precipitation. $x^{\alpha /\beta }$ and $x^{\beta /\alpha }$ are mole fractions of alloying elements on the α and β sides of α/β interface. The values of them were derived from the thermodynamic calculation by using Thermo-Calc^®^ software (2015b) with TTTi3 database. For multicomponent Ti-35421 alloy, *Ω* was evaluated as the weighted summation of the partitioned alloying elements. The Gibbs-Thomson correction to *Ω* was implemented by using the effective temperature *T_eff_* which described the temperature shift due to the Gibbs-Thomson effect.

(A5) $$T_{eff}=T_{eq}\left(1-\frac{\mu_{Ti}^{\beta }-\mu_{Ti}^{\alpha }}{H_{m}}\right).$$

*T_eq_* is the equilibrium transformation temperature; *H_m_* denotes formation enthalpy of α phase in mole; $\mu_{Ti}^{\alpha }$, $\mu_{Ti}^{\beta }$, *H_m_*, and *T_eq_* were all read from the TTTi3 database.

With the lengthening rate *v*, the lengthening distance of the α_lamellar_ tip during the heating can be achieved by

(A6) $$L=\int vdt=\int_{298}^{T}60v\;dT.$$

The integration value *L* is half-length of the α lamella.

### A.2. Dissolution Model of α_lamellar_ Tip

The Aaron equation, Equation (A7) was employed to calculate the average dissolution rate of α_lamellar_ tip.

(A7) $$v=k\Omega \sqrt{\frac{D}{t}}.$$

The supersaturation *Ω* was calculated by Equation (A4) at an effective temperature. By which, the Gibbs-Thomson effect was taken into consideration. In dissolution, both average diffusion coefficients $D^{\alpha }$ of α phase and $D^{\beta }$ of β phases were taken into consideration. The diffusion coefficient of the system followed the equation of

(A8) $$D=D^{\alpha }+D^{\beta }-{\left(D^{\alpha }\right)}^{1-m}{\left(D^{\beta }\right)}^{m}.$$

*m* is the normalized mole fractions between α_lamellar_ and β matrix. The formalism of Equation (A8) was similar to the modeling in the phase-field community [35]. The shrunken distance of single α_lamellar_ tip was obtained by integration Equation (A6) with respect to time/temperature.

### A.3. Thickening Model of α_lamellar_ Broad Face

Due to the ledge mechanism, thickening rate of the broad face is proportional to lengthening rate of the tip with a kinetic coefficient defined as the ratio of ledge height *h* to terrace spacing *b*.

(A9) $$v_{thicken}=\frac{h}{b}v_{lengthen}.$$

The ledge height *h* was set as 2.94 Å based on the atomic diameter of Ti. *b* served as an adjustable parameter between 20 and 50 times of *h* considering that the spacing of terrace changes in the range of 20–50 atoms under high-resolution TEM observation. When calculating *v_lengthen_* with the Ivantsov equation, the last parenthesis of Equation (A1) should be removed, and there was no necessity to calibrate *Ω* by using effective temperature *T_eff_*.

### A.4. Dissolution Model of α_lamellar_ Broad Face below T_eq_

There were two factors contributing to the dissolution of α_lamellar_ broad face, i.e., the compensation flux due to the dissolution of α_lamellar_ tip and thickening of α_lamellar_ broad face. The rate of former followed Equation (A7), while the latter followed Equation (A9). By taking the integrations with respect to temperature, the migration distances of the interface due to the individual factors can be calculated. The migrated distance of α_lamellar_ broad face was the summation of those from two factors.

### A.5. Dissolution model of α_lamellar_ Broad Face above T_eq_ and α_lath_ Broad Face

The dissolution of the broad faces in α_lamellar_ above *T_eq_* and α_lath_ followed simple kinetics of planar interface

(A10) $$v=k\frac{h}{b}\Omega \sqrt{\frac{D}{t}}.$$

*Ω* at the equilibrium state was employed without being calibrated by using *T_eff_*. The diffusion coefficient of the system followed Equation (A8). Additionally, the different values should be assigned to *b* for α_lamellar_ and α_lath_ due to the different spacings of terraces in two α phases.
